# Prognostic value of early proms for one-year recovery trajectories after total hip arthroplasty

**DOI:** 10.1038/s41598-026-39653-7

**Published:** 2026-02-24

**Authors:** Annett Klinder, Frederic Manfred Schrödl, Wolfram Mittelmeier, Martina Rohde-Lindner, Henrike Maria Paulokat, Katrin Osmanski-Zenk

**Affiliations:** 1 Orthopedic Clinic and Policlinic, Rostock University Medical Center, D-18057 Rostock, Germany; 2 Department of Otorhinolaryngology, Head and Neck Surgery “Otto Körner”, Rostock University Medical Center, Rostock, Germany

**Keywords:** Oxford-Hip-Score (OHS), Follow-up, European quality of life 5 dimensions (EQ-5D), EndoCert-Risk-Score (ERS), Rehabilitation, high-risk patients, integrated care, Health services, Quality of life

## Abstract

The improvement of outcome quality and patient satisfaction in total hip arthroplasty (THA) requires a holistic approach. The purpose of this prospective study was to determine whether early postoperative interventions are associated with outcomes one year after THA. A total of 770 patients were assessed for satisfaction and quality of care. Patients were divided into those with and without a physical follow-up examination at the clinic three months postoperatively. Examined patients were subdivided into those with satisfactory or unsatisfactory outcomes. Those with an unsatisfactory 3-month result received targeted therapeutic or diagnostic interventions. Patients with an unsatisfactory clinical examination at three months served as the anchor group to derive a postoperative Oxford Hip Score (OHS) threshold. This threshold was subsequently applied to patients without a physical follow-up examination to identify those at higher risk versus those who had likely achieved a satisfactory outcome. Apart from the OHS, the European Quality of Life 5 Dimensions (EQ-5D) and the EndoCert Risk Score (ERS) were used to determine recovery. Although patients showed progress in rehabilitation and pain reduction, these improvements could not be attributed to the additional measures initiated at the follow-up visit. Intervention measures did not enable patients with unsatisfactory three-month results to reach the outcome levels of those who achieved satisfactory early recovery. Patients with poorer outcomes three months after surgery did not reach comparable PROM levels at twelve months. This finding suggests that early postoperative recovery trajectories are predictive of long-term outcomes. Identifying these patients early and offering targeted multidisciplinary treatment concepts may help improve outcome quality and cost efficiency.

**Supplementary Information:**

The online version contains supplementary material available at 10.1038/s41598-026-39653-7.

## Introduction

The importance of a comprehensive approach to evaluating and improving outcome quality and patient satisfaction in healthcare is well established. The relationship between clinical outcomes and patient-reported satisfaction is complex and multifactorial. In previous studies, our research team explored the link between patient-reported outcome measures (PROMs) and the need for further clinical interventions, aiming to enhance the overall quality of care. Our findings demonstrated that the Oxford Hip Score (OHS) and the EuroQol-5D (EQ-5D) are effective tools for identifying potential high-risk patients as early as three months after surgery. Both assessment tools were assigned specific threshold values, supporting their integration into early screening workflows aimed at initiating timely clinical actions and optimizing outcomes for vulnerable patient groups^1,2^.

Satisfaction rates following hip arthroplasty range between 93% and 96%^3–5^. Nevertheless, in 2023, this implies that approximately 11,000 to 19,000 patients in Germany were dissatisfied with their surgical outcome^6^. While the journal *The Lancet* has referred to hip replacement as the “operation of the century,” it also notes a shift in patient expectations, particularly regarding postoperative physical performance^7^. Furthermore, the quality standards for hospitals continue to rise. The Hospital Transparency Act^8^, which came into effect in March 2024, enables patients to access data on the quality of care provided by hospitals, although the metrics used to assess care quality remain subject to critical discussion.

The German Arthroplasty Registry (EPRD) and the certification process according to EndoCert play a central role in maintaining and continuously enhancing the quality of care, patient safety, and ultimately, outcome quality based on evidence. Both systems aim to involve patients in the care process utilizing “Patient Reported Outcome Measures” (PROMs) for this purpose, which has so far been optional^9–11^.

PROMs are widely used to evaluate outcomes after total hip arthroplasty, but their application and interpretation remain heterogeneous^12^. Although PROMs support quality monitoring, their role in individualized postoperative follow-up and early risk identification is not yet well defined^13^. Recent work highlights that PROM-based alert or monitoring strategies may help detect patients with suboptimal recovery, but evidence regarding their predictive accuracy and clinical usefulness remains limited^14^.

Building upon the findings of our previous study^1^, we refined our study design by incorporating an additional assessment of the established PROMs one year postoperatively.

The aim of this study was to comprehensively analyze and evaluate outcome quality and patient satisfaction based solely on PROMs in comparison to an objective clinical follow-up examination. Due to this aim the following two hypotheses were derived:

1. The PROM scores of patients below the OHS threshold and without clinical examination three months postoperatively (Group 3) differ one year after surgery significantly from those of all other patients.
2. One year postoperatively, the PROM scores of patients who were physically examined at three months and identified as still in need of treatment (Group 2) are comparable —due to additional interventions — to those of patients who had already met the treatment goals at three months (Groups 1 and 4), suggesting that the additional interventions contributed effectively to the recovery process.

## Materials and methods

**Fig. 1:**
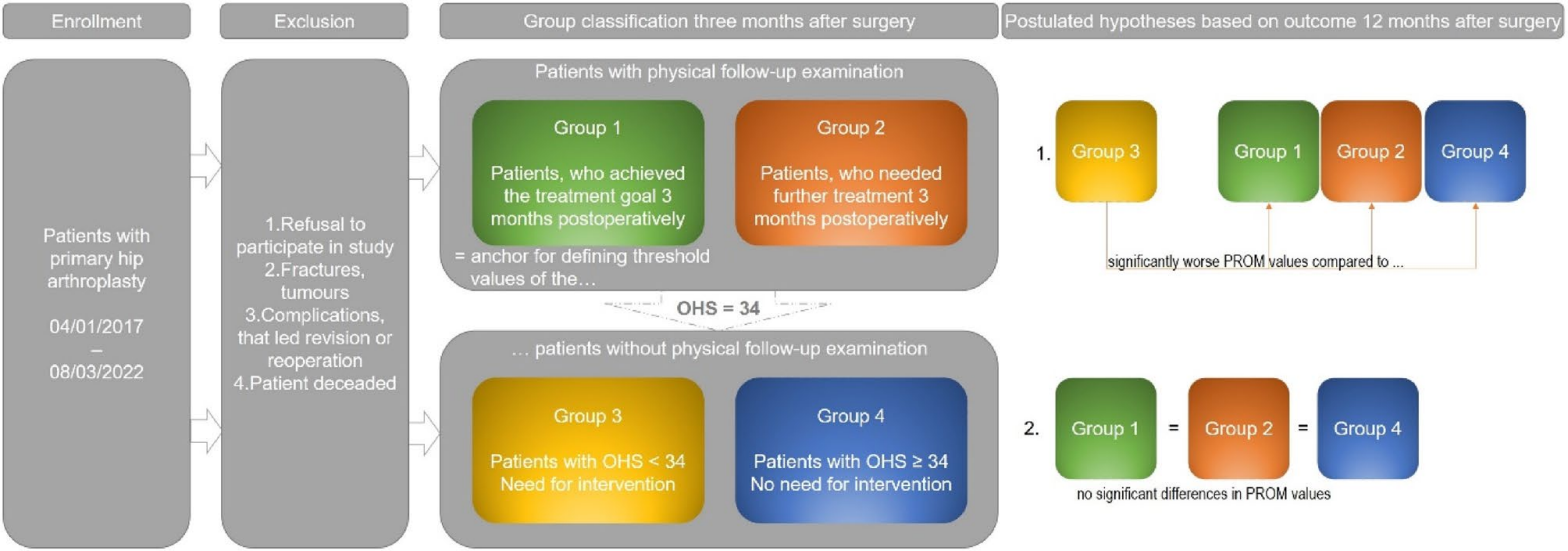
Inclusion and exclusion criteria and group classification and hypotheses of the study; OHS = Oxford Hip Score, PROM = Patient Reported Outcome Measure.

The prospective, observational, single-center study was conducted at the Department of Orthopedic Clinic and Policlinic at Rostock University Medical Center and recruited patients who underwent total hip arthroplasty (THA) between January 2017 and March 2022. Patients who had consented to participate at the time of surgical indication received PROMs at two postoperative time points, with the aim of generating a valid overall assessment of care quality and patient satisfaction. All PROMs, including satisfaction items, were self-administered by the patients at home and returned by mail, ensuring independent and unbiased responses. In addition, a subset of the study population (those who were living within a predefined 25 km radius of the clinic) underwent a clinical follow-up examination after completing the PROM questionnaires three months postoperatively, during which relevant clinical parameters were evaluated to determine the need for further diagnostic and therapeutic intervention by an orthopedic specialist, according to standardized institutional protocols and were supervised by a medical doctoral researcher to ensure consistent data collection^1^. Patients who had already achieved the treatment goal—defined as pain relief and improved joint function—within three months postoperatively were assigned to Group (1) In contrast, patients requiring further interventions, such as physiotherapy, were allocated to Group (2) The results of the clinical follow-up at three months served to define Groups 3 and 4. These two groups consisted of patients who had completed the PROMs at 3 months postoperatively but did not undergo clinical follow-up. Patients in Group 1, who showed no further need for intervention during follow-up, were used as a reference to establish threshold values for the OHS. Accordingly, Group 3 included patients whose OHS fell below this threshold, indicating the potential need for follow-up, while Group 4 comprised those whose scores exceeded the threshold and were therefore considered to have achieved the treatment goal (Fig. 1).

Based on prior analyses conducted by our research group^1^, an a priori power analysis was performed for a one-way analysis of variance (ANOVA) with unequal group sizes (ratio 1:5). From our previous data, we expected that approximately 20% of patients would not reach their treatment goal three months postoperatively. According to our hypothesis that only Group 3 would differ from the other groups twelve months postoperatively, we assumed a mean OHS of 35 for Groups 1, 2, and 4 and a mean of 30 for Group 3, with a pooled standard deviation of 10. Setting the significance level at α = 0.05 and the statistical power at 0.80, the analysis indicated that a total of 576 patients would be required (Group 1 and 4: *n* = 240 each; Group 2 and 3: *n* = 48 each) to detect clinically relevant between-group differences. With a final sample size of 770 patients, the present study thus exceeded the required number of cases, ensuring sufficient statistical power for all analyses.

The study was approved by the local ethics committee (reference number A 2015–0055). For patients who underwent a physical follow-up examination, an additional ethics approval was obtained (A 2016–0146), and a separate informed consent form was signed by these participants on the day of the follow-up examination.

The effects of the interventions initiated three months postoperatively to promote the recovery process were then assessed one year after the surgery to determine the success of the measures taken.

### Patient cohorts and subgroups

**Fig. 2:**
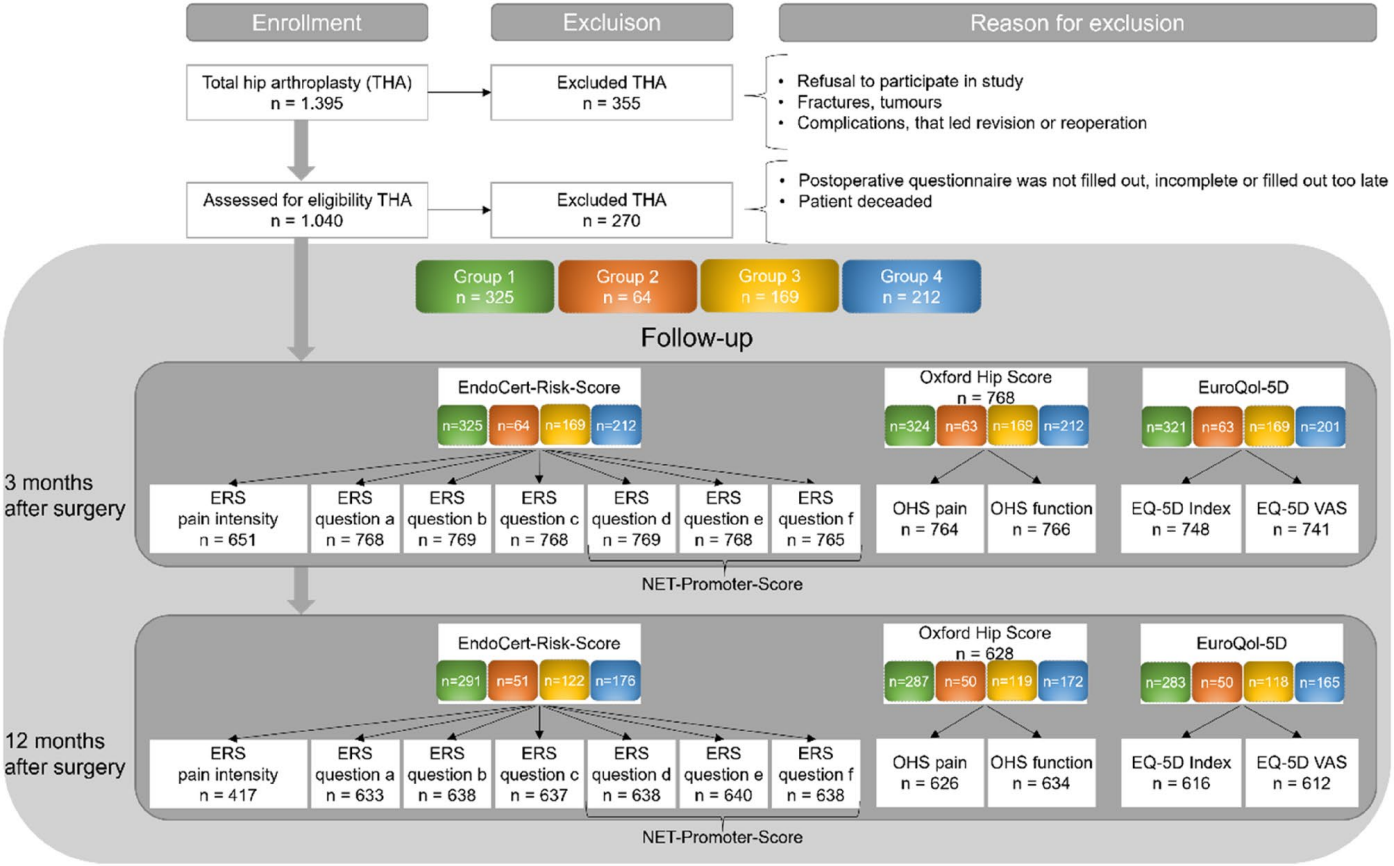
Data on inclusion, exclusion and postoperative course; n = number of cases, THA = Total Hip Arthroplasty, ERS = EndoCert Risk Score: ERS pain intensity = visual analog scale from 0–10, Question a = During the last 4 weeks, have you had pain in your affected joint when walking? Question b = During the last 4 weeks, have you had knee pain at night so that you could not sleep? Question c = Compared to my general state of health over the past 12 months, my current state of health is…? Question d = In hindsight, would you undergo the joint surgery again? Question e = Would you recommend the joint surgery you underwent to another person? Question f = Would you recommend the centre where your joint surgery was performed?, OHS = Oxford Hip Score, EuroQol-5D = European Quality of Life 5 Dimensions – 3 Level Version, VAS = visual analogue scale, Group classification: Group 1: Patients with follow-up examination, who achieved the treatment goal 3 months postoperatively, Group 2: Patients with follow-up examination, who needed further treatment 3 months postoperatively, Group 3: Patients without follow-up examination with OHS < 34 and a potential need of further treatment, Group 4: Patients without follow-up examination with OHS ≥ 34 and no need of further treatment.

During the study period, a total of 1,395 patients underwent THA, which was performed using a standardized anterolateral approach in every case. All surgeries were performed by one of six experienced orthopedic surgeons at a university arthroplasty center. After applying the exclusion criteria as described in the flow chart in Fig. 2, the cohort was reduced to 1,040 patients who had received THA. Following additional exclusions due to deaths or incomplete/unusable questionnaires, a final total of 770 patients with THA were included in the analysis (Fig. 2). Following group allocation, Group 1 comprised 325 patients, Group 2 included 64 patients, Group 3 consisted of 169 patients, and Group 4 contained 212 patients. Mean attrition was slightly higher in the patients who did not undergo a physical examination (Group 3 and 4) compared to the examined patients (Group 1 and 2) with 24% and 16% mean attrition, respectively (Fig. 2). This could be due to the additional contact in the groups undergoing examination^15^.

The PROMs used in this study are standardized and reproducible scoring systems designed to capture a broad range of aspects of daily life. They serve as tools for patients to self-assess their treatment outcomes^16^. Detailed descriptions of the used PROM instruments are provided in the Supplementary Material ^1,17–20^.

### Statistical analysis

The statistical analysis was performed using IBM SPSS Statistics version 29.0 (IBM Corp., New York, USA). Baseline characteristics were compared across the four follow-up groups. Continuous variables with non-normal distribution (age, BMI) are presented as median and range and were analyzed using the Kruskal–Wallis test. Categorical and ordinal variables (sex and ASA) were compared using the Chi-square test. A receiver operating characteristic (ROC) analysis was first conducted to determine the threshold value of the OHS, based on the group of patients who underwent follow-up and required no further intervention (Group 1). Consequently, Group 1, was used as the positive state variable for the ROC analysis to determine the OHS threshold that best discriminated between satisfactory and unsatisfactory postoperative outcomes. The area under the ROC curve (AUC) was used to assess the diagnostic accuracy^21^. Following group allocation, descriptive statistics were calculated for the various PROMs, including median values with minimum and maximum, as well as means with standard deviations, followed by an exploratory data analysis. A two-way ANOVA was conducted to evaluate group-specific differences in PROMs over time, with time and group as independent variables. Statistical significance was set at *p* < 0.05. Sphericity was assessed using Mauchly’s test, and Greenhouse-Geisser correction was applied when sphericity was not fulfilled. Homogeneity of variances was tested using Levene’s test, and depending on the result, either the Bonferroni or Games-Howell post hoc test was applied. Interaction effects between time and group were subsequently analyzed to evaluate their association on the outcomes between the four groups.

## Results

### Receiver operating characteristic analysis (ROC-Analysis)

**Fig. 3:**
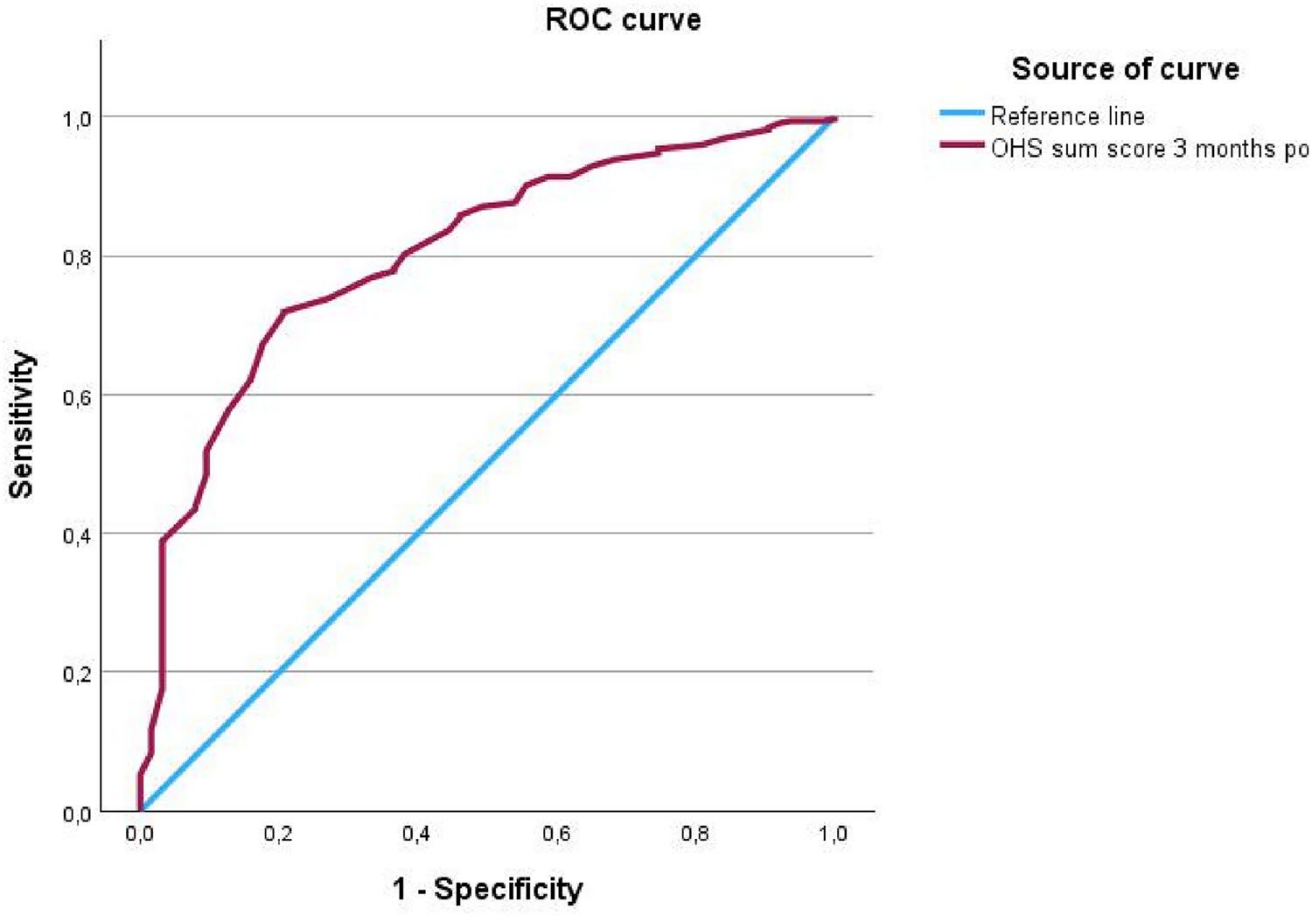
ROC curve based on the OHS sum score of Group 1 and 2 classification.

The ROC analysis for determining threshold value based on three-month postoperative OHS sum score of patients with follow-up examination (*n* = 387) showed good discriminative ability (Fig. 3).

The threshold value as an indicator for the need for follow-up examination resulted in an OHS value of 34. This threshold showed a sensitivity of 71.9% and a specificity of 79.4%, with an AUC of 0.80 (95% CI 0.747–0.859).

### Baseline characteristics

Baseline characteristics did not differ significantly between groups for age, sex, or BMI (Table 1).

**Table 1:** Baseline characteristics by follow-up group. Age, BMI: values are given as median (range). Sex, ASA: values are given as a percentage. *Sex and ASA: Chi-square test; age and BMI: Kruskal–Wallis test.

Variable		Group 1	Group 2	Group 3	Group 4	*p*-value
Age (years)		72 (28–89)	69.5 (23–86)	71 (18–94)	70 (22–89)	0.379
Sex (% female)		53.2	56.3	58	48.6	0.307*
BMI		27.7 (17.69–49.5)	30.5 (19.4–48.7)	37.9 (16.8–45.2)	27.7 (15.8–45.4)	0.141
ASA (in %)	1	10.8	3.1	6.5	9.4	0.137*
	2	61.8	56.3	58.0	60.4	
	3	27.4	40.6	34.3	29.7	
	4	0	0	1.2	0.5	

Group 1: Patients with clinical follow-up, who achieved the treatment goal 3 months postoperatively, Group 2: Patients with clinical follow-up, who needed further treatment 3 months postoperatively, Group 3: Patients without clinical follow-up with OHS < 34, need for intervention, Group 4: Patients without clinical follow-up with OHS ≥ 34, no need for intervention.

### Relationship of the follow-up examination to patient satisfaction

Table 2 provides a detailed overview of the PROM results related to patient satisfaction, presented by the defined study groups as well as overall across all groups. Question c, which assesses changes in general health status over the past 12 months compared to the current health status, revealed that Group 3 (no follow-up, low OHS at 3 months; *p* = 0.033), as well as Group 1 (favorable outcome at 3-month follow-up; *p* = 0.005), underwent significant changes over time. While the condition of Group 1 worsened, that of Group 3 improved. In contrast, Groups 2 and 4 showed no statistically significant changes in response to question c (Group 2: *p* = 0.797; Group 4: *p* = 0.073). Regarding the Net Promoter questions (items d to f), no significant changes over time were observed in any group concerning the willingness to recommend the surgery or the treatment center.

**Table 2:** Descriptive statistics at 3 and 12 months postoperatively and comparison of proms on patient satisfaction over time; median values with different superscript letters are significantly different from each other.

Analyzed parameter	Time point (months post OP)	Group	*N*	Median (Min - Max)	Mean (Standard deviation)	Comparison to 3 months (*p*-value)
EndoCert-Risk-Score question c: Compared to my general state of health over the past 12 months, my current state of health is…	3	1	324	1.00 (1.00–3.00)^a^	1.14 (0.38)	
		2	63	1.00 (1.00–3.00)^b^	1.43 (0.71)	
		3	169	1.00 (1.00–3.00)^b^	1.46 (0.68)	
		4	212	1.00 (1.00–3.00)^a^	1.13 (0.37)	
		Total	768	1.00 (1.00–3.00)	1.23 (0.51)	
	12	1	290	1.00 (1.00–3.00)	1.21 (0.45)	**0.033**
		2	51	1.00 (1.00–3.00)^a^	1.37 (0.63)	0.797
		3	122	1.00 (1.00–3.00)	1.32 (0.59)	**0.005**
		4	174	1.00 (1.00–3.00)^b^	1.17 (0.39)	0.073
		Total	637	1.00 (1.00–3.00)	1.23 (0.49)	
EndoCert-Risk-Score question d: In hindsight, would you undergo the joint surgery again?	3	1	325	1.00 (1.00–4.00)^a^	1.31 (0.63)	
		2	63	1.00 (1.00–4.00)^b^	1.75 (0.93)	
		3	169	1.00 (1.00–5.00)^b^	1.77 (1.09)	
		4	212	1.00 (1.00–4.00)^a^	1.14 (0.48)	
		Total	769	1.00 (1.00–5.00)	1.40 (0.79)	
	12	1	291	1.00 (1.00–5.00)^a^	1.27 (0.65)	0.789
		2	51	2.00 (1.00–4.00)^b^	1.76 (0.95)	0.519
		3	120	1.00 (1.00–5.00)^b^	1.65 (1.07)	0.267
		4	176	1.00 (1.00–4.00)^a^	1.16 (0.49)	0.423
		Total	638	1.00 (1.00–5.00)	1.35 (0.76)	
EndoCert-Risk-Score question e: Would you recommend the joint surgery you underwent to another person?	3	1	324	1.00 (1.00–5.00)^a^	1.29 (0.64)	
		2	63	2.00 (1.00–5.00)^b^	1.75 (0.88)	
		3	169	1.00 (1.00–5.00)^b^	1.72 (1.07)	
		4	212	1.00 (1.00–5.00)^a^	1.22 (0.63)	
		Total	768	1.00 (1.00–5.00)	1.40 (0.80)	
	12	1	291	1.00 (1.00–5.00)^a^	1.29 (0.65)	0.749
		2	51	1.00 (1.00–4.00)^b^	1.65 (0.80)	0.336
		3	122	1.00 (1.00–5.00)^b^	1.57 (0.96)	0.175
		4	176	1.00 (1.00–5.00)^a^	1.21 (0.54)	0.918
		Total	640	1.00 (1.00–5.00)	1.35 (0.72)	
EndoCert-Risk-Score question f: Would you recommend the centre where your joint surgery was performed?	3	1	324	1.00 (1.00–5.00)^a, d^	1.25 (0.60)	
		2	62	1.00 (1.00–5.00)^a, c^	1.55 (0.86)	
		3	167	1.00 (1.00–5.00)^b, c^	1.52 (0.90)	
		4	212	1.00 (1.00–4.00)^d^	1.14 (0.42)	
		Total	765	1.00 (1.00–5.00)	1.30 (0.68)	
	12	1	289	1.00 (1.00–5.00)^a^	1.22 (0.56)	0.459
		2	51	1.00 (1.00–3.00)^a, c^	1.43 (0.67)	0.305
		3	122	1.00 (1.00–5.00)^b, c^	1.45 (0.80)	0.327
		4	176	1.00 (1.00–4.00)^a^	1.16 (0.43)	0.343
		Total	638	1.00 (1.00–5.00)	1.26 (0.60)	

Abbreviations: Group 1: Patients with clinical follow-up, who achieved the treatment goal 3 months postoperatively, Group 2: Patients with clinical follow-up, who needed further treatment 3 months postoperatively, Group 3: Patients without clinical follow-up with OHS < 34, need for intervention, Group 4: Patients without clinical follow-up with OHS ≥ 34, no need for interventionN = number, Min = Minimum, Max = Maximum.

The superscript letters in Table 2 show the comparisons between the four defined groups at each time point. Of particular interest are the results concerning the recommendation of the treating centre (question f). At one year postoperatively, there was no longer a statistically significant difference between Group 2 (patients who underwent follow-up examination, but had not achieved treatment goals at 3 months) and Group 4 (patients without examination who had met treatment goals at 3 months) (*p* = 0.062), in contrast to the significant difference observed at the 3-month mark (*p* = 0.002). Similarly, one year after surgery, there was no significant difference between Group 1 (examined patients who had achieved treatment goals at 3 months) and Group 2 (*p* = 0.183), suggesting that patients from Group 2—despite requiring additional interventions—were just as likely to recommend the arthroplasty center as those in Groups 1 and 4, who had already achieved satisfactory outcomes by 3 months postoperatively.

For the two Net Promoter questions (d and e), no differences were observed either over time or between the groups, as similar results were recorded at both postoperative time points. Consequently, the likelihood of recommending the procedure to others, as well as the willingness to undergo the surgery again, remained consistently stable throughout the study period.

One year postoperatively, a significant difference in self-assessed health status compared to the previous year (question c) was found only between patients who underwent clinical follow-up and required intervention (Group 2) and those without examination who had already achieved treatment success (Group 4) (*p* = 0.047). A comparison between Group 3 (non-examined patients with presumed need for intervention) and Group 4 showed a p-value of 0.062, all other group comparisons also failed to reach statistical significance. The alignment of outcomes in Group 2 (patients requiring intervention at three months postoperatively) with those of Group 1 (patients who had already achieved treatment goals at that time) highlights the effectiveness of the postoperative interventions that were implemented. At the same time, the notable improvement in Group 3 (non-examined patients with initially poor OHS scores) should be acknowledged, despite the absence of targeted clinical interventions. Additionally, the observed convergence between Group 1 (examined patients with good OHS at 3 months) and Group 2 (examined patients with poor OHS at 3 months) may partly be attributed to the significant decline in health status within Group 1 over time (*p* = 0.109).

### Relationship of the follow-up examination to pain

Table 3 summarizes the results of the pain-related PROMs for each study group as well as for the overall cohort. Additionally, the table illustrates changes in pain perception between the two postoperative time points. While the OHS pain subscore showed a statistically significant improvement over time in all four groups (Groups 1, 2 and 3: *p* < 0.001; Group 4: *p* = 0.032), the single-item measure of overall pain intensity did not reflect significant changes in patients that achieved the treatment goal at three months (Group 1: *p* = 0.595) or the non-examined group without expected need for intervention (Group 4: *p* = 0.9239). Further insight is provided by the ERS pain-related questions. Question a, assessing pain while walking, showed significant improvement in all groups (Groups 1 & 3: *p* < 0.001; Group 2: *p* = 0.024; Group 4: *p* = 0.019). Similarly, question b, evaluating pain at night, showed significant reductions in pain in Groups 1, 2, and 3 (Groups 1 & 3: *p* < 0.001; Group 2: *p* = 0.004), while Group 4 did not show a statistically significant change (*p* = 0.227). These findings indicate that even patients who were already considered successfully treated at the three-months follow-up (Groups 1 and 4) still continued to experience reductions in specific types of pain—namely during walking and at night.

**Table 3:** Descriptive statistics at 3 and 12 months postoperatively and comparison of proms on pain over time; median values with different superscript letters are significantly different from each other.

Analyzed parameter	Time point (months post OP)	Group	*N*	Median (Min - Max)	Mean (Standard deviation)	Comparison to 3 months (*p*-value)
EndoCert-Risk-Score pain intensity	3	1	305	2.00 (0.00–10.00)^a^	2.24 (2.01)	
		2	61	4.00 (0.00–9.00)^b^	4.28 (2.30)	
		3	142	4.00 (0.00–10.00)^b^	4.25 (2.38)	
		4	143	2.00 (0.00–8.00)^a^	2.22 (1.73)	
		Total	651	3.00 (0.00–10.00)	2.86 (2.27)	
	12	1	173	2.00 (0.00–10.00)^a^	2.22 (2.24)	0.595
		2	43	3.00 (0.00–8.00)^a, c^	3.21 (2.10)	**0.015**
		3	96	3.00 (0.00–10.00)^b, c^	3.24 (2.53)	**< 0.001**
		4	105	1.00 (0.00–9.00)^a^	1.91 (2.15)	0.923
		Total	417	2.00 (0.00–10.00)	2.28 (2.33)	
EndoCert-Risk-Score question a: During the last 4 weeks, have you had pain in your affected joint when walking?	3	1	325	2.00 (1.00–4.00)^a^	1.80 (0.66)	
		2	63	2.00 (1.00–4.00)^b^	2.38 (0.85)	
		3	169	2.00 (1.00–4.00)^b^	2.23 (0.86)	
		4	211	2.00 (1.00–4.00)^c^	1.60 (0.60)	
		Total	768	2.00 (1.00–4.00)	1.89 (0.76)	
	12	1	291	1.00 (1.00–4.00)^a^	1.52 (0.67)	**< 0.001**
		2	51	2.00 (1.00–4.00)^b^	2.06 (0.84)	**0.024**
		3	121	2.00 (1.00–4.00)^b^	1.93 (0.85)	**< 0.001**
		4	170	1.00 (1.00–4.00)^a^	1.45 (0.66)	**0.019**
		Total	633	1.00 (1.00–4.00)	1.62 (0.75)	
EndoCert-Risk-Score question b: During the last 4 weeks, have you had hip pain at night so that you could not sleep?	3	1	325	1.00 (1.00–4.00)^a^	1.36 (0.57)	
		2	63	2.00 (1.00–4.00)^b^	1.90 (0.80)	
		3	169	2.00 (1.00–4.00)^b^	1.92 (0.83)	
		4	212	1.00 (1.00–4.00)^a^	1.29 (0.48)	
		Total	769	1.00 (1.00–4.00)	1.51 (0.69)	
	12	1	291	1.00 (1.00–3.00)^a^	1.20 (0.46)	**< 0.001**
		2	50	1.00 (1.00–4.00)^b^	1.56 (0.76)	**0.004**
		3	122	1.00 (1.00–4.00)^b^	1.52 (0.70)	**< 0.001**
		4	175	1.00 (1.00–4.00)^a^	1.20 (0.53)	**0.027**
		Total	638	1.00 (1.00–4.00)	1.29 (0.57)	
Oxford Hip Score: Subscore pain	3	1	323	79.17 (8.33–100.00)^a^	75.78 (19.28)	
		2	63	54.17 (12.50–100.00)^b^	52.98 (23.29)	
		3	168	45.83 (0.00–83.33)^c^	46.18 (18.30)	
		4	210	83.33 (58.33–100.00)^d^	84.17 (9.85)	
		Total	764	75.00 (0.00–100.00)	69.70 (22.78)	
	12	1	285	91.67 (20.83–100.00)^a^	86.78 (16.67)	**< 0.001**
		2	51	75.00 (8.33–100.00)^b^	69.40 (25.25)	**< 0.001**
		3	120	79.17 (0.00–100.00)^b^	72.05 (23.38)	**< 0.001**
		4	170	91.67 (12.50–100.00)^a^	88.16 (16.09)	**0.032**
		Total	626	91.67 (0.00–100.00)	82.91 (20.08)	

Abbreviations: Group 1: Patients with clinical follow-up, who achieved the treatment goal 3 months postoperatively, Group 2: Patients with clinical follow-up, who needed further treatment 3 months postoperatively, Group 3: Patients without clinical follow-up with OHS < 34, need for intervention, Group 4: Patients without clinical follow-up with OHS ≥ 34, no need for intervention, N = number, Min = Minimum, Max = Maximum.

The comparative analysis of the four defined groups at each postoperative time point is presented in Table 3 using superscript letters. This presentation allows for a direct comparison of group results and illustrates the differences and similarities between the groups at the different assessment time points. In the individual evaluation of pain intensity, statistically significant differences were observed three months postoperatively between examined patients requiring intervention (Group 2) and those without intervention needs (Group 1), as well as between examined patients requiring intervention (Group 2) and non-examined patients without an expected need for intervention (Group 4) (*p* < 0.001). However, at the one-year postoperative time point, these groups no longer showed statistically significant differences (Group 1 vs. Group 2: *p* = 0.073; Group 2 vs. Group 4: *p* = 0.093).

### Relationship of the follow-up examination to function and quality of life

**Fig. 4:**
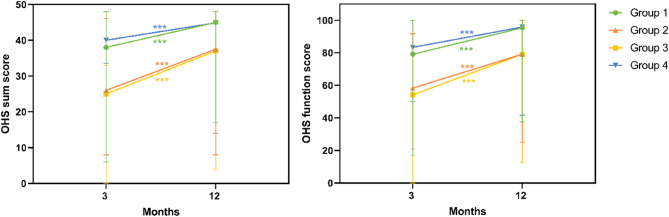
Descriptive statistics at 3 and 12 months postoperatively and comparison of PROMs on function over time.

Figure 4 presents the results of the PROMs assessing function while Table 4 shows those of quality of life. The data are reported for the predefined study groups and as total value of all groups. Additionally, Fig. 4; Table 4 illustrate the time course of function and quality of life throughout the study period, as well as comparisons between the four groups at each time point.

Overall, both the Oxford Hip Score total and the Oxford Hip function subscore improved significantly in all groups one year postoperatively compared to three months after surgery (*p* < 0.001) (Fig. 4). Group comparisons at each time point showed that all four groups differed significantly at 3 months postoperatively. At 12 months, Groups 1 and 4 demonstrated significantly higher scores compared with Groups 2 and 3, while no significant differences were observed between Groups 1 and 4 or between Groups 2 and 3. The EQ-5D-VAS, which reflects the patient’s self-rated current health status, showed a significant improvement over time in examined patients without need for further intervention (Group 1) and in non-examined patients with an expected need for intervention (Group 3) (Group 1: *p* = 0.039, Group 3: *p* = 0.004). In contrast, no significant changes were observed in Groups 2 and 4 (Group 2: *p* = 0.350, Group 4: *p* = 0.926). Regarding the EQ-5D-index, only Group 3 demonstrated a statistically significant improvement over time (*p* < 0.001), while quality of life remained unchanged in Group 1 (*p* = 0.088), Group 2 (*p* = 0.103), and Group 4 (*p* = 0.563) (Table 4).

**Table 4:** Descriptive statistics at 3 and 12 months postoperatively and comparison of proms on quality of life over time; median values with different superscript letters are significantly different from each other.

Analyzed parameter	Time point (months post OP)	Group	*N*	Median (Min - Max)	Mean (Standard deviation)	Comparison to 3 months (*p*-value)
European Quality of Life 5 Dimensions visual analogue scale	3	1	321	80.00 (3.00–100.00)^a^	76.57 (17.95)	
		2	63	60.00 (10.00–95.00)^b^	62.22 (19.32)	
		3	158	65.00 (0.00–99.00)^b^	61.60 (21.38)	
		4	199	82.00 (8.00–100.00)^a^	81.03 (16.43)	
		Total	741	80.00 (0.00–100.00)	73.35 (20.01)	
	12	1	280	89.00 (3.00–100.00)^a^	80.12 (19.29)	**0.039**
		2	48	77.50 (2.00–100.00)^b^	67.13 (26.59)	0.350
		3	118	70.00 (5.00–100.00)^b^	67.59 (23.10)	**0.004**
		4	166	85.00 (5.00–100.00)^a^	80.80 (20.58)	0.926
		Total	612	80.00 (2.00–100.00)	76.87 (21.77)	
European Quality of Life 5 Dimensions -Index	3	1	321	0.887 (0.020–1.000)^a^	0.887 (0.160)	
		2	63	0.788 (0.110–1.000)^b^	0.707 (0.235)	
		3	163	0.788 (−0.200–1.000)^b^	0.717 (0.227)	
		4	201	0.999 (0.260–1.000)^a^	0.930 (0.106)	
		Total	748	0.887 (−0.200–1.000)	0.846 (0.194)	
	12	1	283	1.000 (0.260–1.000)^a^	0.916 (0.127)	0.088
		2	50	0.844 (0.110–1.000)^b^	0.796 (0.234)	0.103
		3	118	0.800 (0.020–1.000)^b^	0.801 (0.211)	**< 0.001**
		4	165	1.000 (0.180–1.000)^a^	0.937 (0.112)	0.563
		Total	616	0.999 (0.020–1.000)	0.890 (0.164)	

Abbreviations: Group 1: Patients with clinical follow-up, who achieved the treatment goal 3 months postoperatively, Group 2: Patients with clinical follow-up, who needed further treatment 3 months postoperatively, Group 3: Patients without clinical follow-up with OHS < 34, need for intervention, Group 4: Patients without clinical follow-up with OHS ≥ 34, no need for intervention, N = number, Min = Minimum, Max = Maximum.

## Discussion

The two initial hypotheses – first, that PROM outcomes in patient group 3 would differ significantly from those of the other groups at one year postoperatively, and second, that PROM outcomes in Group 2 would align with those of groups 1 and 4 as a result of early postoperative interventions following physical examination – were largely not supported by the data. The results indicate that patients who had not reached the defined treatment goals within three months after surgery tended to not achieve comparable levels of functional recovery and pain relief at the one-year mark, even when targeted therapeutic interventions were provided. In contrast, patients demonstrating satisfactory clinical outcomes at the three-month follow-up maintained superior PROM results over the course of the study. Consequently, we believe that early postoperative PROMs at three months strongly predicted one-year outcomes. As this study was observational in nature, causal relationships between follow-up interventions and postoperative outcomes cannot be established. The findings indicate associations rather than causation. However, unlike many studies deriving PROM-based thresholds solely from score distributions, our analysis incorporated a clinician-established reference standard^22^. The 3 months specialist examination provided an independent assessment of treatment success, which served as the anchor for deriving the OHS threshold. This process strengthens the internal validity of the threshold within our cohort. But, as thresholds for PROM-based risk stratification may vary across institutions and patient populations, external validation in an independent dataset remains necessary before considering broader clinical implementation.

Overall, the four groups exhibited significant improvements in pain and function measures, even in the groups that had already met the treatment goal three months postoperatively. Thus, the longitudinal development of PROM outcomes in the defined patient groups was consistent with findings from previous studies^23,24^. Harris et al.^22^ described a so-called treatment failure (TF) at one year postoperatively with an OHS value below 25.5. On average, all groups in this study were already above this value one year after THA, and thus did not fall below the TF threshold. Therefore, the treatment goal defined by Harris et al. was achieved in all groups one year postoperatively, whether or not interventions were performed^22^. This should be taken into consideration when discussing the hypotheses.

The first hypothesis must be rejected for all individual questions and scores, as Groups 2 and 3 remained nearly identical in all scores and individual questions one year postoperatively. The non-examined patient group with poor scores three months postoperatively (Group 3) did not differ at the 12-month from the patient group (Group 2) who received further therapeutic interventions during the clinical follow-up. Both groups showed significant improvement over time. Therefore, it may be assumed that Group 3 received the necessary therapies during their outpatient visits to their local orthopedist, which explains the continuous improvement in their health status. Therefore, clinic-based follow-up did not demonstrate a clear additional benefit for patients. Our second hypothesis was based on previous studies, which have shown that follow-up measures such as medical rehabilitation, physiotherapy, or IRENA (Intensified Rehabilitation Aftercare), a structured outpatient program offered by the German Pension Insurance following inpatient rehabilitation, can lead to improvements in postoperative complaints^25–29^. The success of the intervention was therefore evaluated one year postoperatively using validated PROMs. It was expected that Groups 1, 2, and 4 would show homogeneous outcomes one year after surgery, whereas Group 3, by contrast, would differ significantly. However, this hypothesis could not be confirmed for functionality, whereas with regard to pain intensity and the likelihood of recommending the clinic, the second hypothesis was valid. Our findings demonstrate that, in contrast to the outcomes observed at 3 months postoperatively, there were no longer any significant differences in pain intensity or clinic recommendation rates between patients who required intervention after clinical follow-up (Group 2) and those who had already achieved the treatment goal by 3 months postoperatively (Groups 1 and 4) at the 1-year time point. However, the expected long-term benefits of clinical follow-up examination and subsequent treatment for patients with poorer postoperative scores at 3 months were not fully realized by 12 months postoperatively. In particular, in terms of the joint-specific Oxford Hip Score, including both subscores, as well as the health-related quality of life measured by the EQ-5D, both hypotheses must be refuted. Patients who, according to clinical assessment, had not achieved the treatment goal at the 3-month postoperative examination (Group 2) and subsequently received additional interventions, did not reach the same outcome scores at 12 months as those who had already met the treatment goal at 3 months (Groups 1 and 4). Considering the 3-month comparison, in which patients without physical examination but with poor OHS scores (Group 3) had significantly worse results in the overall Oxford score and the Oxford pain subscore than patients with physical examination and indicated need for further intervention (Group 2), it becomes evident that Group 3 improved more notably over time. Moreover, quality of life, as measured by the EQ-5D-Index, remained unchanged over time in Groups 1, 2, and 4, while Group 3 showed significant improvement, even slightly exceeding the index level of Group 2. This development may be attributed to various patient characteristics that were not further stratified within the scope of this study^30^ and underlines the limited impact of the clinic-based follow-up.

Enhanced recovery and structured clinical pathways have been shown to accelerate early postoperative improvement and reduce variability in outcomes, yet they also consistently report a subgroup of patients who fail to achieve expected early gains despite optimized care^31^. Postoperative care following joint arthroplasty should prioritize sustained recovery, particularly in patients at risk of treatment failure. As demonstrated by Osmanski-Zenk et al.^1^ the need for rehabilitation follow-up or further interventions can be assessed based on threshold values of the OHS. Furthermore, the findings of the present study may indicate that a physical examination at a specialized clinic is not a requirement for evaluating postoperative outcomes, as care provided in outpatient settings may appear to be equally effective. Contrary to concerns raised by Greitemann et al.^32^ regarding the lack of information on adherence to follow-up care recommendations, the results of this study show significant improvements in all groups—even in the absence of targeted clinical follow-up examination. Patients commonly engage with private practice physicans and outpatient specialists not solely for symptomatic treatment, but also for routine preventive care^33^. This outpatient-based approach offers several advantages: patients are typically under long-term specialist care, and physicians are familiar with the patient’s clinical course and social environment. Kaiser Permanente in the United States has optimized this approach by implementing perioperative care pathways and comprehensive postoperative follow-up through dedicated case managers^34^. Larger medical care centers in Germany are already developing comparable integrated care structures. Fast-track surgery is one such concept that proved effective by not only shortening hospital stays, but also contributing to lower rates of morbidity and mortality, enhanced postoperative mobility, and increased patient satisfaction^35^. PROMs in fast-track settings have shown comparable or even superior results relative to conventional approaches^36^, however, some studies report higher revision rates for THA^37^. International data provide strong evidence of the cost-efficiency and clinical benefits of outpatient care^38^. The concept of short-stay fundamental and transitional care could function as an intermediary between inpatient treatment and outpatient follow-up services^39–41^. The elimination of the strict separation between inpatient and outpatient care based on overnight stays is currently under discussion^38^. So far the successful implementation of these schemes is often rated according to length of stay, postoperative complications and short-term recovery, however, a few studies reported medium-term follow-ups for up to 12 months, also including the use of PROMs^42–44^. Our study shows that PROM-derived threshold values provide a valuable tool to identify those patients, who, despite coordinated care and additional consultations, demonstrate delayed recovery in the advanced phase of rehabilitation, i.e. beyond 12 weeks after THA.

### Limitations of this study

1. As this study is observational, the results describe associations rather than causal effects. The grouping based on the clinical follow-up examination reflects clinical decision-making rather than a randomized or interventional assignment.
2. One major limitation of this study is the lack of consideration for patients’ preoperative comorbidities and other confounders. Comorbidities negatively impact PROMs, as joint-specific symptoms can be difficult to distinguish from those caused by other health issues. This is particularly relevant at the one-year follow-up, where no clinical examination takes place and the data relies solely on patients’ subjective reports. The absence of adjustment for comorbidities and other confounders therefore introduce bias into the results. Future studies should systematically assess comorbidities and incorporate them into the analysis. In addition, other potential confounders such as BMI, opioid use, and preoperative disease severity were not systematically collected and therefore could not be analyzed, which further limit the interpretation of postoperative satisfaction and PROM outcomes.
3. Another limitation of this study is the insufficient follow-up regarding the implementation of targeted therapeutic interventions. Any interventions received by Group 3 remain unknown. Without this information, a valid interpretation of the results is limited, as it is not clear which additional factors beyond the primary intervention may have affected the treatment outcomes. Future studies should prospectively record the type, frequency, and setting of such interventions to allow a more valid interpretation of their potential effects on treatment outcomes.
4. Because follow-up visits were performed by one of the operating surgeons rather than by an independent assessor, observer bias cannot be fully excluded. However, standardized documentation and the supervision by a medical doctoral researcher likely reduced its influence.
5. Although all eligible patients within a predefined geographic radius (25 km) as well as those who returned the questionnaires within three months, were systematically invited for the 3-month clinical follow-up, and the vast majority attended, a small and unquantified proportion may not have presented. Therefore, a minor selection bias cannot be entirely excluded. However, given the standardized invitation procedure and the very high attendance rate, we consider its impact on group composition to be minimal.

## Conclusion and outlook

Although early interventions were initiated by the operating clinic at three months postoperatively for patients who had not yet achieved the treatment goal, their outcomes at one year postoperatively did not reach the level of those patients who had already demonstrated satisfactory recovery at three months. Patients with lower OHS at the three-month mark who were neither re-examined nor received follow-up treatment in the clinical setting achieved comparable results at the one-year follow-up. In order to improve outcomes for these notable cases, it will be necessary to define high-risk groups more precisely and to implement comprehensive preoperative preparation strategies for these patients. Standardized indication criteria should be established and applied to facilitate the identification and selection of high-risk individuals.

A central reporting system, such as the EPRD, could be used to automatically notify patients who fall below defined PROM thresholds at three months, prompting further therapeutic interventions. Electronic health records and intelligent networking systems would support this process and promote patient engagement in line with the goals of the EPRD and EndoCert initiatives.

## Supplementary Information

Below is the link to the electronic supplementary material.

Supplementary Material 1

## Data Availability

The data presented in this study are available upon request from the corresponding author. The data are not publicly available, but can be obtained from the Department of Clinical Research at the Orthopedic Department of the University Medicine Rostock if required.
