# Supplementary material for: Prognostic value of early proms for one-year recovery trajectories after total hip arthroplasty
Source: Sci Rep. 2026 Feb 24;16:7508. doi: 10.1038/s41598-026-39653-7 (PMC12932692; doi:10.1038/s41598-026-39653-7)
Supplement: Supplementary file 1 — Supplementary Material 1 [file 41598_2026_39653_MOESM1_ESM.docx]

# **Supplement**

# **Descriptions of the PROMs**

The six-component EndoCert Risk Score (ERS) assesses pain intensity, overall health status, and satisfaction with the joint surgery through the NET Promoter Score (questions d-f) [1]. The latter includes response options regarding whether the patient would undergo the surgery again, recommend it, and recommend the arthroplasty center, with five response options ranging from "definitely yes" to "definitely not." In the evaluation, responses 1 are considered promoters, 2 are passive, and responses 3 to 5 are categorized as detractors. [2]. In addition, the Oxford Hip Score (OHS) was recorded, a validated PROM instrument consisting of 12 questions answered on a 5-point Likert scale. The OHS evaluates pain, function, and the ability to perform daily tasks related to the operated hip joint. The responses were summed to a total score, with a maximum of 48 points, where higher values indicate better outcomes: scores over 41 are considered excellent, 34-41 are good, 27-33 are satisfactory, and scores below 27 are deemed unsatisfactory [3]. Missing responses were replaced by the mean score of the answered questions, as long as no more than two questions were unanswered. The OHS can be divided into pain and function subscores, with pain being represented by questions 1, 8, 9, 10, 11, and 12, and function by questions 2-7. These subscores can each reach a maximum value of 100, with 0 representing the worst possible outcome [4]. In addition, the European Quality of Life 5 Dimensions – 3 Level Version (EQ-5D-Index) was administered, a generic PROM used to assess overall health status and quality of life. This questionnaire comprises five dimensions: mobility, self-care, usual activities, pain/discomfort, and anxiety/depression, each with three response options ("no problems," "some problems," "extreme problems"). Additionally, patients rated their health status using a visual analog scale (EQ-5D VAS) ranging from 0 (very poor) to 100 (very good). The design of the EQ-5D-3L followed the German valuation algorithm [5].

References

1. Osmanski-Zenk, K. *et al.* Identification of Potential High-Risk Patients on the Basis of PROMs in a Certified Centre for Joint Replacement (EndoProthetikZentrum) Using the Example of Hip Arthroplasty. A Proof-of-Concept Study Regarding the Utilization of PROMs. *Zeitschrift fur Orthopadie und Unfallchirurgie* **160,** 442–454 (2022).

2. Osmanski-Zenk, K., Ellenrieder, M., Mittelmeier, W. & Klinder, A. Net Promoter Score: a prospective, single-centre observational study assessing if a single question determined treatment success after primary or revision hip arthroplasty. *BMC musculoskeletal disorders* **24,** 849 (2023).

3. Murray, D. W. *et al.* The use of the Oxford hip and knee scores. *The Journal of bone and joint surgery. British volume* **89,** 1010–1014 (2007).

4. Harris, K. K. *et al.* Can pain and function be distinguished in the Oxford Hip Score in a meaningful way? : an exploratory and confirmatory factor analysis. *Bone & joint research* **3,** 305–309 (2014).

5. Greiner, W., Claes, C., Busschbach, J. J. V. & Schulenburg, J.-M. G. von der. Validating the EQ-5D with time trade off for the German population. *The European journal of health economics : HEPAC : health economics in prevention and care* **6,** 124–130 (2005).
